# Thirty-Day Readmission Rates in Orthopedics: A Systematic Review and Meta-Analysis

**DOI:** 10.1371/journal.pone.0123593

**Published:** 2015-04-17

**Authors:** James T. Bernatz, Jonathan L. Tueting, Paul A. Anderson

**Affiliations:** Department of Orthopedics and Rehabilitative Medicine, University of Wisconsin School of Medicine and Public Health, Madison, Wisconsin, United States of America; University of Michigan, UNITED STATES

## Abstract

**Background:**

Hospital readmission rates are being used to evaluate performance. A survey of the present rates is needed before policies can be developed to decrease incidence of readmission. We address three questions: What is the present rate of 30-day readmission in orthopedics? How do factors such as orthopedic specialty, data source, patient insurance, and time of data collection affect the 30-day readmission rate? What are the causes and risk factors for 30-day readmissions?

**Methods/Findings:**

A review was first registered with Prospero (CRD42014010293, 6/17/2014) and a meta-analysis was performed to assess the current 30-day readmission rate in orthopedics. Studies published after 2006 were retrieved, and 24 studies met the inclusion criteria. The 30-day readmission rate was extrapolated from each study along with the orthopedic subspecialty, data source, patient insurance, time of collection, patient demographics, and cause of readmission. A sensitivity analysis was completed on the stratified groups. The overall 30-day readmission rate across all orthopedics was 5.4 percent (95% confidence interval: 4.8,6.0). There was no significant difference between subspecialties. Studies that retrieved data from a multicenter registry had a lower 30-day readmission rate than those reporting data from a single hospital or a large national database. Patient populations that only included Medicare patients had a higher 30-day readmission rate than populations of all insurance. The 30-day readmission rate has decreased in the past ten years. Age, length of stay, discharge to skilled nursing facility, increased BMI, ASA score greater than 3, and Medicare/Medicaid insurance showed statistically positive correlation with increased 30-day readmissions in greater than 75 percent of studies. Surgical site complications accounted for 46 percent of 30-day readmissions.

**Conclusions:**

This meta-analysis shows the present rate of 30-day readmissions in orthopedics. Demonstrable heterogeneity between studies underlines the importance of uniform collection and reporting of readmission rates for hospital evaluation and reimbursement.

## Introduction

Recent proposed changes in hospital and physician reimbursement place greater emphasis on reducing adverse events associated with surgical procedures [1]. The Centers for Medicare and Medicaid Services (CMS) as well as private insurance agencies scrutinize the costs of these complications, and they have highlighted readmission within 30 days of hospital discharge as a potential area for improvement [2,3]. The Affordable Care Act added a section to the Social Security Act establishing a Hospital Readmissions Reduction Program. This required the CMS to reduce payments to hospitals with excess 30-day readmissions following acute myocardial infarction, heart failure and pneumonia [4]. This pilot program is expanding to include chronic obstructive pulmonary disease and total hip and knee replacement in 2014 [5]. Thus, it is paramount to understand the current rates of readmission to help establish a benchmark at which hospitals can be monetarily penalized.

Readmission rates reported in the literature have varied depending on the patient profile and specialty of service. Within orthopedics alone, reported 30-day readmission rates have ranged from 2–14% [6,7]. Given the wide range of patient age, medical comorbidity, and procedure complexity within orthopedics, this variability is not unexpected. However, to our knowledge, the literature lacks any systematic reviews on the 30-day readmission rates reported for different subspecialties within orthopedics.

The goal of this analysis is to first understand the incidence of 30-day readmissions in orthopedics. From there, we will examine factors associated with 30-day readmission as well as frequent causes of readmission. This study aims to find: a) thirty-day readmission rates stratified by subspecialty (spine, arthroplasty, trauma, and general); b) how factors such as orthopedic specialty, data source, patient insurance, and time of data collection affect the 30-day readmission rate; and c) the causes and risk factors of 30-day readmission.

## Materials and Methods

### Electronic Literature Search

**Fig 1:**
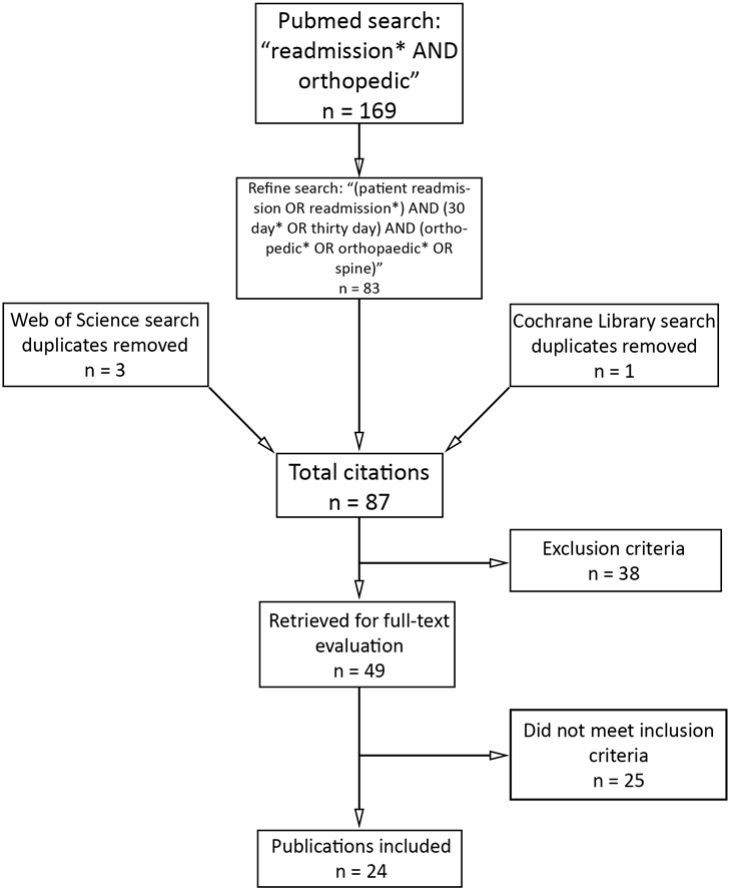
Flowchart of systematic review methodology.

The study was registered with Prospero, an international database of prospectively registered systematic reviews (CRD42014010293, 6/17/2014). Two independent reviewers then conducted a systematic literature search of four electronic databases (PubMed, Web of Science, Cochrane Library, and Google Scholar) for articles published in English after 2006 (Fig 1). Searches were performed using Medical Subject Headings (MeSH) used by the National Library of Medicine. The MeSH terms were used to produce the search: “(patient readmission OR readmission*) AND (30 day* OR thirty day) AND (orthopedic* OR orthopaedic* OR spine).” This search yielded 83 articles from PubMed. Four additional, non-duplicate studies were found using the same search criteria in the Web of Science and the Cochrane Library. Google Scholar did not yield any additional studies. These 87 articles underwent title/abstract review and 38 met the primary exclusion criteria. Studies were eliminated if: the study tested a specific medical device, surgical technique, or post-operative care protocol (18 studies), the patients were already subgrouped (i.e. diabetic patients with hip fracture) (9), the majority were outpatient procedures (3), there were fewer than 100 patients (3), the study did not report on orthopedic procedures/admissions (3), or if the data collection began before the year 2000 (2) (S1 Table). This left 49 studies for full-text review. The inclusion criteria were studies that quantified 30-day readmission rates following any orthopedic procedure or admission; twenty-five did not report an all-cause 30-day readmission rate. This left 24 publications included in this study. There were no restrictions on the study design (retrospective, prospective, cohort, case-control, etc.). Studies that included inpatient-only procedures or both inpatient and outpatient procedures were considered.

### Data Extraction

The following data were extracted by a single author: sample, readmissions within 30 days, subspecialty, cause of readmission, risk factors for readmission, data source, date of enrollment, inpatient versus outpatient procedures, and tracking of admissions to outside hospitals. This was done by reading of the full article with interpretation of figures and tables. Causes of readmission were pooled from studies that reported these data. We collected risk factors that were reported by univariate or multivariate analysis when available. Statistically positive correlation with 30-day readmission or no statistical correlation was recorded for all risk factors reported. Risk factors that were examined in only one study were not analyzed. The time of data collection, which we refer to as “date of enrollment,” was grouped into three categories of four year increments: after 2009, between 2006 and 2009, and before 2006. A “30-day readmission” was defined as an admission to any service of any hospital within 30 days of either an orthopedic procedure or discharge from the orthopedic service. The data source was categorized as: single hospital’s database, multicenter registry database (collecting data on 2–15 hospitals), or large national database (Center for Medicare and Medicaid Services (CMS), Veteran Affairs (VA), and National Surgical Quality Improvement Program (NSQIP)). Patient information included age, gender, smoking status, body mass index (BMI), American Society of Anesthesiologists (ASA) score, Charlson Comorbidity Index (CCI), and All Patient Refined Diagnosis Related Groups Severity of Illness Score (APR-DRG SOI). All quantitative values were recorded as mean and standard deviation. Patient insurance status was grouped into three categories: VA, Medicare, or other. Studies that accepted all insurances were defined as “unrestricted.” Gender was reported as proportion of males in the total patient population.

### Bias & Quality Assessment

All studies were assessed for bias according to the Quality in Prognosis Studies (QUIPS) tool [40]. The tool contains six domains assessing (1) participation, (2) attrition, (3) prognostic factor measurement, (4) outcome measurement, (5) confounding, and (6) statistical analysis and reporting. All studies were given a rating of low, moderate, or high risk of bias for each domain based on the prompting items and considerations outlined in the tool. If a domain could not be applied to a study, “NA” was recorded. Additionally, each study was given a prognostic level of evidence according to the Journal of Bone and Joint Surgery guidelines [41].

The risk factors were evaluated for quality of evidence using the adjusted Grading of Recommendations Assessment, Development and Evaluation (GRADE) framework [42]. Each risk factor was evaluated according to the seven GRADE factors and was given a score of having no serious limitations or serious limitations based on study bias. The risk factors that had five or more scores of ‘no serious limitations’ were determined to be high quality while those that had three or four scores of ‘no serious limitations’ were determined to be moderate quality. Risk factors with fewer than three scores of ‘no serious limitations’ were determined to be low quality.

### Data Analysis

Comprehensive Meta-Analysis, version 2.2050 (Biostat, Englewood, NJ, USA) was used for data pooling. Sensitivity analysis was performed by sequential removal of all studies from the analysis. To assess historical changes and determine a correlation between the date of patient enrollment and 30-day readmission rate, we used a method of moments meta-regression.

All confidence intervals (CI) were reported at 95 percent. P-value statistical significance was measured at 0.05. Heterogeneity was assessed by calculating the Cochrane Q-value and the I-squared statistic. They are both quantitative measures of the amount of heterogeneity between pooled studies. A Q-value closer to the degrees of freedom signifies low heterogeneity. I-squared has an upper limit of 100%. I-squared values of 25%, 50% and 75% were considered indicative of low, moderate and high heterogeneity, respectively. The studies were assumed to be heterogeneous. Funnel plots were used to assess publication bias. Sensitivity analysis was performed by single elimination of each study and determining if statistical results were changed.

## Results

### Systematic Review

We identified 24 studies reporting 30-day readmissions in orthopedic surgery (Fig 1). All were retrospective observational studies. From the 24 studies we are reporting on 26 unique patient populations as two of the studies reported two separate populations based on the subspecialty [22,24]. The total number of patients included was 487,780, with studies ranging from 412 to 343,068 patients (Table 1). Five studies included non-operative admissions [7,21,26,27,29]. These studies did not report which of the readmissions were from non-operative admissions, therefore this could not be analyzed.

**Table 1:** Evidentiary table.

Citation	Specialty	Insurance	Data Source	Data Collection	Patients	Readmissions (%)	Weight**(%)
Cullen, ARCSE 2006[8]	THA	All	Single hospital	Aug 1997—Mar 2001	769	65 (8.5)	4.02
Vorhies, CORR 2012[9]	TKA	Medicare	CMS	Jan 2002—Dec 2007	4057	228 (5.7)	4.58
Vorhies, JArth 2011[10]	THA	Medicare	CMS	Jan 2002—Dec 2007	1809	123 (6.8)	4.37
Wang, Spine 2012[11]	Spine	Medicare	CMS	Jan 2003—Dec 2007	343068	27102 (7.9)	4.83
Zmistowski, JBJS 2013[12]	Arthroplasty	All	Single hospital	Jan 2004—Dec 2008	10633	348 (3.3)	4.67
Morris, JAMASurg 2014[13]	Arthroplasty	VA	VA	Jan 2005—Dec 2009	2273	175 (7.7)	4.50
Schairer, CORR (a) 2014[14]	THA	All	Single hospital	Jan 2005—Dec 2011	1415	61 (4.3)	4.36
Schairer, CORR (b) 2014[15]	TKA	All	Single hospital	Jan 2005—Dec 2011	1408	56 (4.0)	4.36
Cram, MCP 2012[16]	TKA	Medicare	CMS	Jan 2006—Dec 2006	64712	5320 (8.2)	4.82
Schairer, Spine 2013[7]	Spine	All	Single hospital	Jan 2006—Dec 2011	836	116 (13.9)	4.31
Hoyer, JHM 2014[17]	All Ortho	All	Single hospital	Jul 2006—Dec 2012	3292	273 (8.3)	4.61
McCormack, Spine 2012[18]	Spine	All	Single hospital	Jan 2007—Dec 2009	3673	156 (4.2)	4.47
Amin, JNSS 2013[19]	Spine	All	Single hospital	Oct 2007—Jun 2011	5780	281 (4.9)	4.63
Dailey, JBJSAm 2013[20]	All Ortho	All	Single hospital	Jul 2008—Jun 2010	3261	137 (4.2)	4.43
Hageman, JOT 2014[21]	Trauma	All	Single hospital	Jan 2008—Dec 2011	3452	186 (5.4)	4.52
Bosco, JArth 2014[22]	THA	Medicare	Single hospital	Jan 2009—Dec 2012	1077	57 (5.3)	4.34
Bosco, JArth 2014[22]	TKA	Medicare	Single hospital	Jan 2009—Dec 2012	1263	55 (4.4)	4.34
Clement, JArth 2013[23]	THA	All	Single hospital	Jul 2009—Jun 2011	1583	103 (6.5)	4.30
Mesko, JArth 2014[24]	All Ortho	All	Single hospital	May 2010—Apr 2011	2368	159 (6.7)	4.55
Mesko, JArth 2014[24]	Arthroplasty	All	Single hospital	May 2010—Apr 2011	1291	46 (3.6)	4.55
Kim, JNSS 2014[25]	Spine	All	NSQIP	Jan 2011—Dec 2011	7016	314 (4.5)	4.65
Lovecchio, Spine 2014[26]	Spine	All	NSQIP	Jan 2011—Dec 2011	2320	59 (2.6)	3.99
Basques, Spine 2014[27]	Spine	All	NSQIP	Jan 2011—Dec 2012	2339	87 (3.7)	4.22
Pugely, Spine 2014(a) [28]	Spine	All	Multicenter	Jan 2012—Dec 2012	2005	79 (3.9)	4.17
Pugely, Spine 2014(b) [29]	Spine	All	Multicenter	Jan 2012—Dec 2012	15668	695 (4.4)	4.75
Issa, JKS 2014[6]	TKA	All	Multicenter	*	412	8 (2.0)	1.90
Total	-	-	-	-	487780	36083	100.00

* Not reported

** Random effects model

(a) and (b) are 2 separate publications that share the same author, journal, and publication year

All studies were retrospective observational studies

Readmissions = 30-day readmissions. THA = Total Hip Arthroplasty. TKA = Total Knee Arthroplasty. VA = Veterans Affairs. CMS = Center for Medicare & Medicaid Services. NSQIP = National Surgical Quality Improvement Program.

Fifteen of the 26 study populations were limited to arthroplasty (six THA, six TKA, and three included all joints combined), nine spine, three all orthopedic admissions, and one trauma. Nineteen of the groups had no restriction on patient insurance, while six included only Medicare beneficiaries, and one was Veteran Affairs. Fifteen studies were reports of data from a single hospital, three from multicenter registries, and eight from national databases (four CMS, three NSQIP, one VA) (Table 1).

### Thirty-Day Readmissions

**Fig 2:**
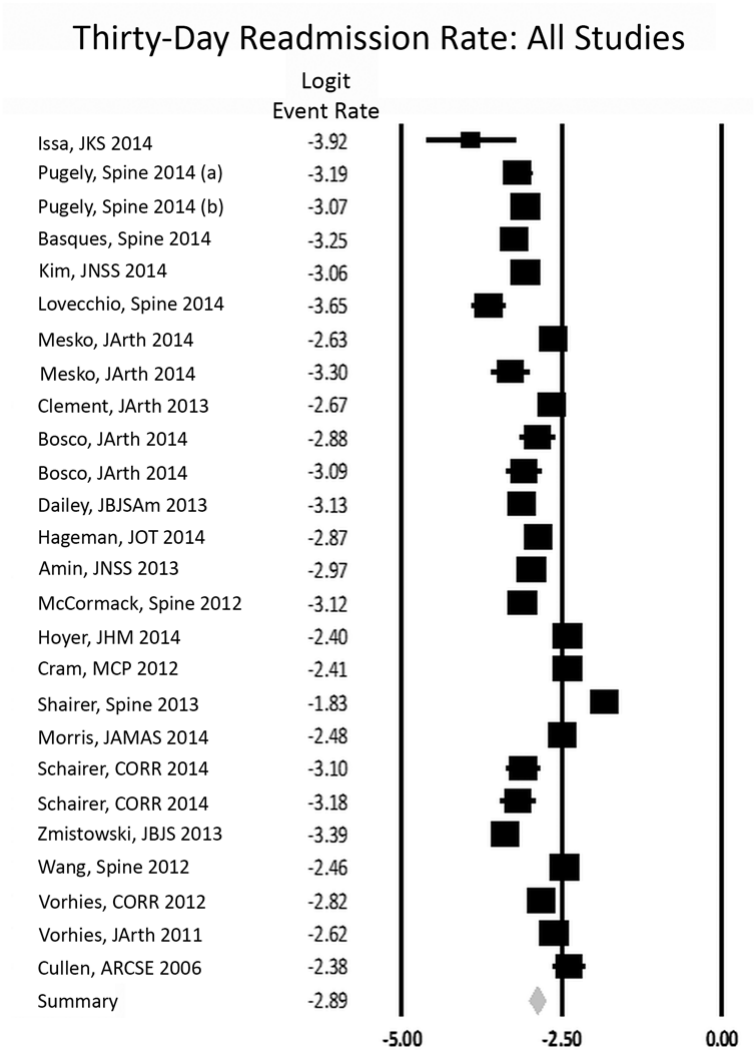
Forest plot of the natural log of the 30-day readmission rate with 95% confidence interval. Logit event rate = natural log of 30-day readmission rate.

**Fig 3:**
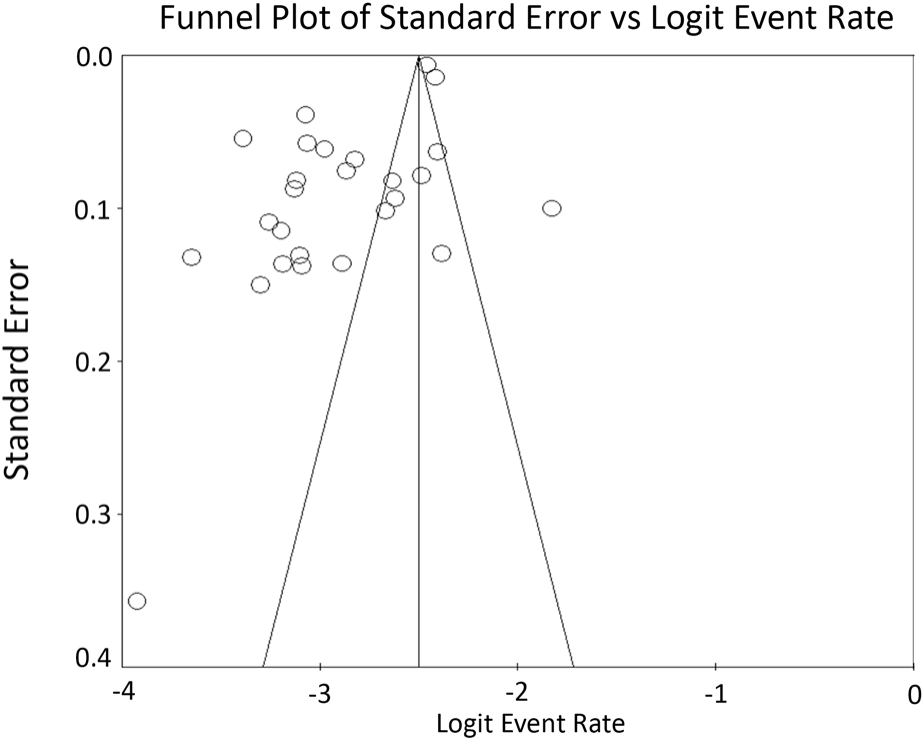
Funnel plot assessing publication bias. Missing studies to the right of average and near the bottom indicate that the literature lacks studies with higher readmission rates and fewer patients, respectively. Logit event rate = natural log of 30-day readmission rate.

The 30-day readmission rate across all orthopedic specialties was 5.4% (Confidence interval: 4.8,6.1) (Fig 2). The studies had high heterogeneity with an I-squared value of 98.2%. The funnel plot for publication bias shows that there are studies missing to the right of average, meaning that there is a lack of studies with a higher readmission rate (Fig 3). There are also few studies near the bottom of the funnel, suggesting the literature is missing studies with fewer patients. Studies testing for publication bias including Orwin’s fail safe and the trim and fill did not indicate that missing studies would have changed the results significantly. Sensitivity analysis did not change any outcomes significantly.

### Subspecialty 30-Day Readmissions

**Fig 4:**
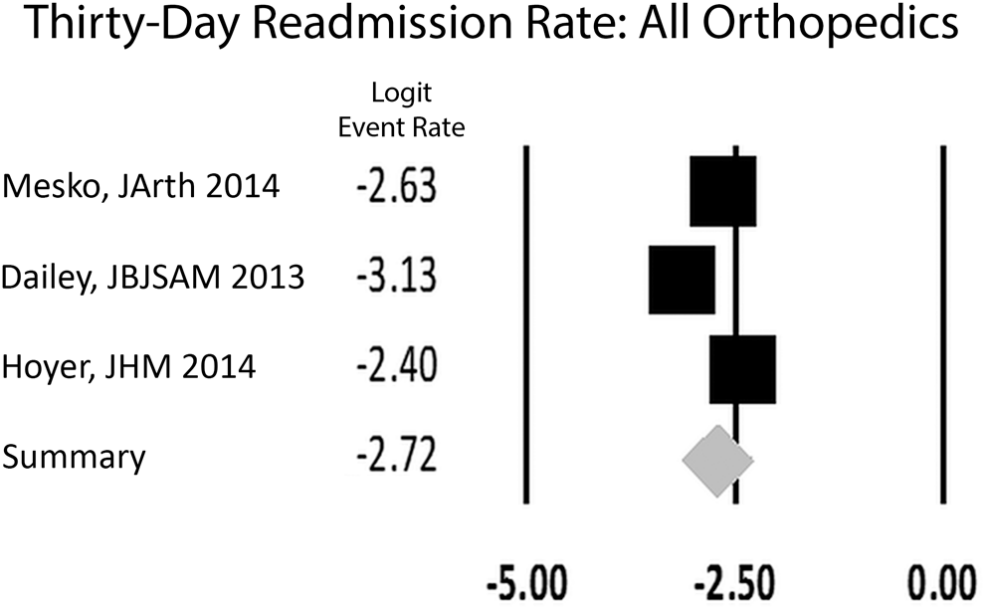
Forest plot for studies examining all orthopedic subspecialties. Logit event rate = natural log of 30-day readmission rate.

**Fig 5:**
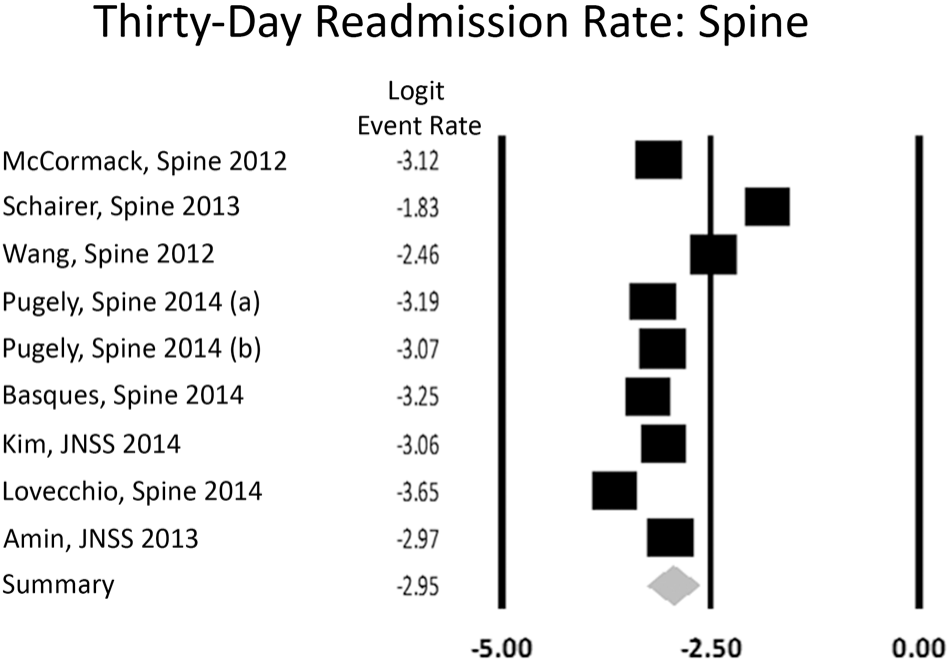
Forest plot for studies examining spine. Logit event rate = natural log of 30-day readmission rate.

When stratified by subspecialty, spine had a rate of 5.0% (CI: 3.7,6.6), combined arthroplasty group 5.2% (CI: 4.1,6.6), and all orthopedics 6.2% (CI: 4.2,9.1) (Figs 4 and 5). This was not statistically significant (p = 0.84, Table 2). All orthopedics included the three studies that reported a single readmission rate for all orthopedic procedures. The single trauma study reported a 30-day readmission rate of 5.4% [21].

**Table 2:** Thirty-Day Readmission Rates Stratified by Subspecialty.

Subspecialty	Studies	Patients	Readmission (%)*	CI Lower	CI Upper	Q	I-squared (%)
**All Orthopedics**	3	8921	6.2	4.2	9.1	45.1	95.6
**Spine**	9	382705	5.0	3.7	6.6	678.4	98.8
**Arthroplasty**	13	92702	5.2	4.1	6.6	442.0	97.5
**Trauma**	1	3452	5.4	-	-	-	-
**Total**	26	488780	5.4	4.8	6.0	-	-

CI = 95% Confidence Interval.

*No difference present between all orthopedics, spine, arthroplasty, and trauma, p-value = 0.835.

**Fig 6:**
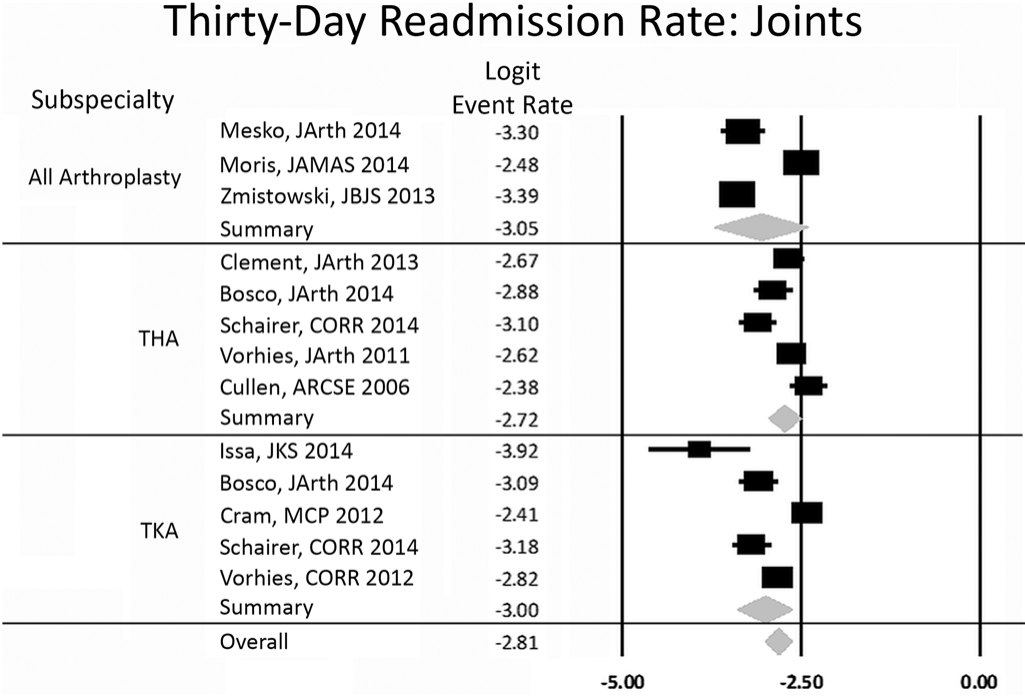
Forest plot for arthroplasty studies. Logit event rate = natural log of 30-day readmission rate.

We compared populations that reported THA, TKA, and combined arthroplasty (total shoulder arthroplasty, TKA, TKA). THA had a rate of 6.2%, TKA 4.8%, and combined 4.5% (Fig 6). There was no statistically significant difference (p-value = 0.68, Table 3).

**Table 3:** Arthroplasty 30-Day Readmission Rates Stratified by Subspecialty.

Subspecialty	Studies	Patients	Readmission (%)*	CI Lower	CI Upper	Q	I-squared (%)
**All Joints**	3	14197	4.5	2.4	8.3	90.6	97.8
**THA**	5	6653	6.2	5.0	7.5	18.1	77.9
**TKA**	5	71852	4.6	3.3	6.8	103.8	96.2
**Total**	13	92702	4.6	3.1	7.4	-	-

CI = 95% Confidence Interval.

*No difference present between all joints, THA, and TKA, p-value = 0.679.

### Comparison Between Data Sources

The three studies using a multicenter clinical registry had the lowest 30-day readmission rate at 3.9% (Table 4). This rate is lower than the national databases’ rate of 5.6% and the single hospital database rate of 5.8%. The p-value for this analysis was 0.020, representing statistical significance.

**Table 4:** Thirty-Day Readmission Rates Stratified by Data Source.

Data Source	Studies	Patients	Readmission (%)*	CI Lower	CI Upper	Q	I-squared (%)
**Single Hospital**	11	39761	5.8	4.5	7.3	300.3	96.7
**Multicenter**	3	18085	3.9	3.1	5.0	6.5	69.3
**National**	9	429934	5.6	5.0	6.3	313.6	97.5
**Total**	24	487780	5.3	4.8	5.9	-	-

Studies with two patient populations collapsed into one. CI = 95% Confidence Interval.

*Statistically significant difference present between all single hospital, multicenter, and national database, p-value = 0.0198.

### Patient Insurance

Medicare patients showed a significantly higher 30-day readmission rate of 6.9% when compared to populations with unrestricted insurance at 4.5% (p-value = 0.000037, Table 5). The single study including only VA patients reported a rate of 7.7%.

**Table 5:** Thirty-Day Readmission Rates Stratified by Patient Insurance.

Insurance	Studies	Patients	Readmission (%)*	CI Lower	CI Upper	Q	I-squared (%)
**Unrestricted**	16	66229	4.5	4.0	5.6	278.5	94.6
**Medicare**	5	415986	6.9	6.4	7.5	71.2	94.4
**VA**	1	2273	7.7	-	-	-	-
**Total**	22	484,488	6.4	5.9	6.9		

Studies with two patient populations collapsed into one. CI = 95% Confidence Interval.

*Statistically significant difference present between unrestricted, Medicare, and VA insurance, p-value = 0.0000367.

### Effect of Enrollment Date

**Fig 7:**
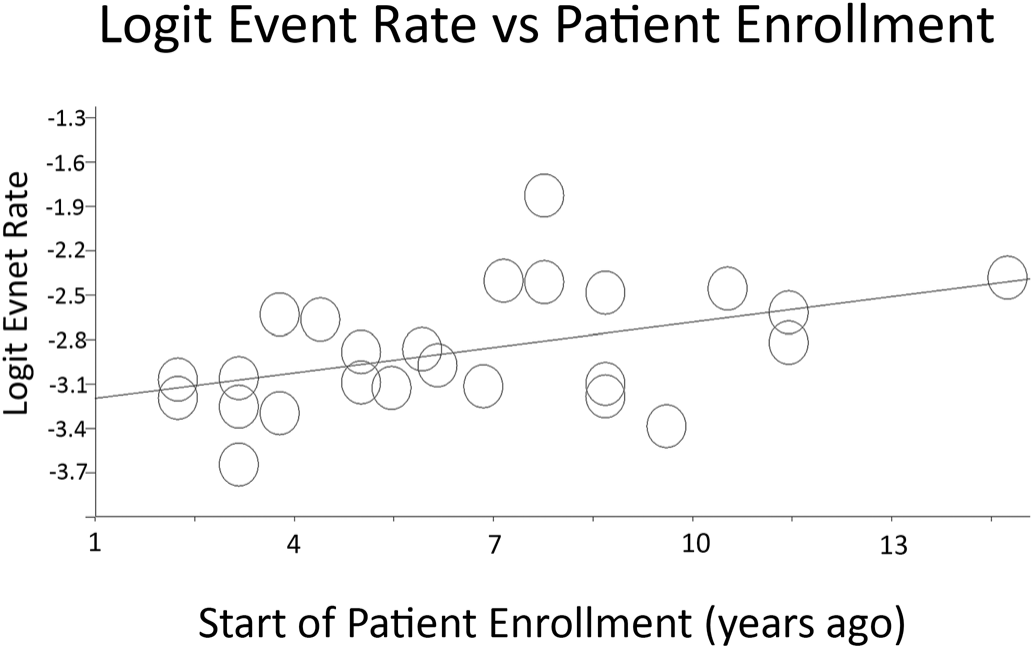
Regression plot of 30-day readmission rate versus time when patient enrollment began. Regression line: y = 0.004x – 3.3. P-value = 0.00292. Logit event rate = natural log of 30-day readmission rate.

The most recent studies, which began collecting data after 2009, had a 30-day readmission rate of 4.5% (Table 6). This is significantly lower than the 30-day readmission rate of 7.7% in studies that began data collection between 2006–2009 and the 7.8% readmission rate demonstrated in studies that began data collection before 2006 (p-value = 0.030). When plotted as the natural log of 30-day readmission rate versus time since enrollment began, the meta-regression was statistically significant with p-value of 0.0029 (Fig 7).

**Table 6:** Thirty-Day Readmission Rates Stratified by Time of Data Collection.

Enrollment Date	Studies	Patients	Readmission (%)*	CI Lower	CI Upper	Q	I-squared (%)
**After Jan 1, 2010**	6	365432	4.5	4.3	4.8	49.9	88.0
**Jan 1, 2006 to Dec 31, 2009**	9	88929	7.7	7.5	7.9	300.7	97.7
**Before Dec 31, 2005**	8	33007	7.8	7.7	7.9	366.9	98.4
**Total**	22	487368	7.6	7.5	7.7	-	-

Studies with two patient populations collapsed into one^22,24^. One study excluded for lack of data. CI = 95% Confidence Interval.

*Statistically significant difference present between the three time periods, p-value = 0.0303.

### Causes of Readmission

We identified 10 causes of readmission that were reported in three or more studies. Meta-analysis yielded surgical site infection as the most common cause of 30-day readmission at 32.2% (Table 7). Combined with non-infectious wound problems, which accounted for 14.0% of readmissions, surgical site complications were the cause of 46.2% of 30-day readmissions. Medical problems occurred in 26.4% of readmissions, and specifically DVT had a rate of 3.5%. Surgical problems accounted for the fewest 30-day readmissions, with dural tear, fixation failure, and pain summing to 21.2% of 30-day readmission causes.

**Table 7:** Pooled Causes of Readmission.

Category	Cause	Number of Studies Reporting	Citations	Number of Patients Readmitted With Diagnosis	Percentage of Readmissions (%)	CI Lower	CI Upper
**Wound-related**	**SSI**	11	7,11,14,15,18,19,20,21,23,27,29	4771	32.2	22.7	43.4
	**Non-Infectious Wound Problem**	9	7,12,14,15,18,19,20,20,27	232	14.0	9.1	21.0
	**Hematoma**	7	7,11,14,15,18,23,29	1028	4.2	3.3	5.2
	**Seroma**	4	7,14,15,29	49	6.5	4.0	10.4
	**Cellulitis**	3	14,15,23	23	9.0	1.9	34.0
**Surgical**	**Dural Tear**	4	7,18,19,29	45	4.1	2.3	7.3
	**Fixation Failure**	4	7,18,21,29	83	9.4	4.0	20.5
	**Pain**	6	14,18,20,23,27,29	188	7.7	3.4	16.3
**Medical**	**Medical Complication**	6	7,11,14,18,26,29	1136	26.4	18.9	35.5
	**DVT**	5	14,18,20,23,29	49	3.5	1.3	9.0

SSI = Surgical Site Infection. DVT = Deep Venous Thrombosis. CI = 95% confidence interval

### Risk Factors for Readmission

Twenty-two risk factors were identified that were examined on univariate or multivariate analysis in at least two studies (Table 8). The risk factors positively associated with increased 30-day readmissions were: age (8), length of hospital stay (7), discharge to skilled nursing facility (4), increased BMI (3), and ASA score greater than 4 (3). Diabetes, male sex, and history of pulmonary disease were found to have no correlation in greater than 50 percent of the studies reporting data. Protective factors associated with decreased odds of readmission could not be analyzed because they were included in only one study. The GRADE analysis demonstrated that two of the identified risk factors (age and length of stay) were high quality based on minimal bias from the reporting studies. Six studies were of moderate quality and the remaining fourteen risk factors were low quality.

**Table 8:** Risk Factors for Readmission with GRADE Analysis.

				GRADE Factors							
Risk Factor	Patient Populations With Statistically Positive* Correlation (Citations)	Patient Populations With No Correlation (Citations)	Patient Populations Not Reporting	Study Limitations	Inconsistency	Indirectness	Imprecision	Publication Bias	Moderate/Large Effect Size	Dose Effect	Overall Quality
**Age**	8 (11^†^,17,21,23^†^,24^†^,27,29)	2 (25,26)	14	✓	✖	✖	✓	✓	✓	✓	+++
**Length of Stay**	7 (12,15,20,23^†^,24^†^,29)	1 (7)	16	✓	✖	✖	✓	✓	✓	✓	+++
**Discharge to SNF**	4 (7,15,24^†^)	1 (12)	19	✓	✖	✖	✓	✖	✓	✖	++
**Increased BMI**	3 (12,23^†^,27)	0	21	✓	✖	✖	✓	✖	✓	✓	++
**ASA ≥ 4**	3 (25,28,29)	0	21	✓	✖	✓	✓	✖	✓	✖	++
**Medicare/Medicaid**	3 (11,24^†^)	1 (20)	20	✓	✖	✖	✓	✖	✖	✖	+
**Operative Duration**	3 (25,28,29)	4 (7,24^†^,26)	17	✓	✖	✓	✓	✖	✖	✓	++
**Length of Stay in ICU**	2 (14,20)	0	22	✓	✖	✖	✖	✖	✖	✓	+
**Black or African-American**	2 (11^†^,20)	0	22	✓	✖	✖	✓	✖	✖	✖	+
**American Indian or Alaskan Native Race**	2 (20,29)	0	22	✓	✖	✖	✓	✖	✖	✖	+
**Cardiac Valve Disease**	2 (7,14)	0	22	✓	✖	✖	✖	✖	✖	✖	+
**Revision**	2 (14,15)	0	22	✓	✖	✖	✖	✖	✓	✖	+
**Blood Transfusion**	2 (24^†^)	0	22	✓	✖	✖	✖	✖	✖	✖	+
**CCI**	2 (11^†^,21)	1 (12)	21	✓	✖	✖	✓	✖	✓	✓	++
**Diabetes**	2 (7,14)	3 (25–27)	19	✓	✖	✖	✖	✖	✖	✓	+
**Male Sex**	2 (12,17)	5 (11u,23^†^,24^†^,27)	17	✓	✖	✓	✓	✓	✖	✖	++
**ASA 3 or 4**	1 (27)	1 (25)	22	✓	✖	✖	✖	✖	✖	✖	+
**Steroid Use**	1 (27)	1 (25)	22	✓	✖	✖	✖	✖	✖	✖	+
**Heart Disease**	1 (7)	1 (27)	22	✓	✖	✖	✖	✖	✖	✖	+
**Anemia**	1 (25)	1 (7)	22	✓	✖	✖	✓	✖	✖	✖	+
**Recent Weight Loss**	1 (29)	1 (7)	22	✓	✖	✖	✓	✖	✖	✖	+
**Pulmonary Disease**	1 (29)	3 (7)	20	✓	✖	✖	✓	✖	✖	✖	+

*P-value ≤ 0.05 on multivariate analysis unless noted by †

^†^ indicates univariate analysis

SNF = Skilled Nursing Facility. BMI = Body Mass Index. ICU = Intensive Care Unit. CCI = Charlson Comorbidity Index.

GRADE Factors: ✓, no serious limitations; ✖, serious limitations (or not present for moderate/large effect size, dose effect). Overall quality of evidence: +, low; ++, moderate; +++, high

### Quality Assessment

The results of the QUIPS quality assessment are presented in S2 Table. The greatest risk of bias was seen in the study participation domain. Twelve studies were determined to have a high risk of bias because they reported only on a subset of orthopedic patients (i.e. spine) and reported data only from a single hospital. Both of these factors contribute to a lack of generalizability for the results of the study. All studies were determined to have a low risk of bias in the outcome measurement and statistical analysis domains because all studies reported 30-day readmissions and the total number of eligible patients. All studies were retrospective cohort studies and thus qualified as prognostic level III.

### Heterogeneity

The assumption that all studies were heterogeneous was confirmed by the high Q-values and I-squared values that were consistently greater than 90 percent, indicating that greater than 90 percent of the variance was due to differences between studies.

## Discussion

Ultimately, our aim is to initiate interventions to lower the frequency of 30-day readmissions. The main goal of this study is to determine the present 30-day readmission rate in orthopedics. Our meta-analysis shows that the 30-day readmission rate across all orthopedic specialties is between 4.8 and 6.0 percent. Given the high heterogeneity, it is best to consider the confidence interval rather than the pooled result (5.4 percent). The Center for Medicare & Medicaid Services reports the same value for specifically hip and knee surgeries [5]. The orthopedic readmission rate in absolute terms is 7–9 percent lower than rates reported in meta-analyses of general internal medicine and six percent lower than general surgery [30,31]. This 30-day readmission rate could be underreported, as patients may be treated at a different hospital within 30 days and may not be reflected in our study. In our experience, the rate of 30-day readmission to outside hospitals has been difficult to identify. On the other hand, we have found that 5.4 percent may overstate unplanned readmissions by almost 25 percent by including planned readmissions for staged procedures. In general the data was collected using International Classification of Diseases, Ninth Revision (ICD-9) coding as well as Current Procedural Terminology (CPT) data, which have both been shown to have inaccuracies [32,33].

The 5.4 percent 30-day readmission rate we report for orthopedics is encouraging compared to other specialties but reveals an opportunity for further improvement. There are known risk factors for orthopedic 30-day readmissions such as age, length of stay, discharge to skilled nursing facility, BMI, and ASA score [11,13,18,20,21,23,25,27,29], which were confirmed in our study. Based on the GRADE analysis, age and length of stay were the two risk factors with the highest quality of supporting evidence. In the majority of manuscripts in this current study, these risk factors were either not reported or were presented in a way that is not adequate for quantitative analysis. Thus, there is a need to report this data and stratify outcomes based on risk factors to allow for pooling and improved statistical power. Furthermore, to our knowledge, no studies have implemented or reported on quality improvement initiatives to lower the 30-day readmission rate based on this knowledge of risk factors. This is an important knowledge gap that warrants future study.

Examination of the readmission causes highlights potential targets for 30-day readmission reduction. Nearly half of all readmissions were due to wound complications, the majority of which were surgical site infections. There have been many strategies directed at infectious complications, such as compliance with antibiotics [34], screening programs for methicillin-resistant *Staphylococcus aureus* [35], decolonization [36], and intraoperative optimization of air quality [37]. Non-infectious wound problems, including hematoma, seroma, and cellulitis, however, present a different clinical problem. Although these diagnoses may point toward prevention through improvement of surgical techniques (wound closure and intraoperative hemostasis), their treatment is less well defined and standardized. Better definitions, prevention, and treatment strategies for non-infectious wound problems could reduce 30-day readmission rates. In particular, wounds that are not found to be infected and do not require intravenous antibiotics could potentially be managed in the outpatient setting. Medical complications were, in our opinion, relatively low but improved patient optimization prior to surgery could have an impact on reducing 30-day readmissions. It should be noted that risk factors and causes of readmission were missing from greater than 75 percent of studies and thus the conclusions drawn represent only what is available in published literature.

There was significant heterogeneity between studies based on Q and I-squared values. This is not surprising given the variable populations and procedures encountered in surgical diseases. Heterogeneity suggests that important covariates between studies may have a significant influence on outcomes. The number of patients, patient age, insurance, subspecialty, and type of procedure all varied between studies. Additionally, the inclusion of non-operative patients, outpatient surgeries, and admissions to outside hospitals was inconsistent. Even further, variable patient age and insurance suggest that the populations may have had baseline comorbidity differences. Although we could not analyze many of these variables due to inconsistent reporting in the studies, we believe that the heterogeneity could be driven by these factors and suggest exploration of these factors in future studies.

When stratified by subspecialty, 30-day readmission rates ranged from 4.5–6.2%. Although these differences were not statistically significant, they may represent a clinically significant difference. For example, quality improvement efforts may be better directed at total hip arthroplasty (THA) versus spine procedures, as THA has a larger margin for improvement. The mean direct cost for a readmission following THA has been reported at 17,000 dollars by Bosco et al. [22] and by 2030, the demand for primary THA in the United States is estimated to grow by 174 percent to 572,000 [38]. Thus, decreasing the 30-day readmission rate of joint arthroplasty to the frequency currently achieved by spine or trauma could equate to millions of dollars in saved cost. We attempted multivariate analysis by subspecialty (i.e. arthroplasty readmission rates reported by single institution vs. multicenter registry vs. national registry), however there was insufficient data. This could be a direction for future studies.

The three studies using multicenter registry databases showed a lower 30-day readmission rate than single hospital or national databases. Although this was statistically significant, we believe this to be confounded by the subspecialties in these three studies. One of the studies examined only a specific type of cruciate-sparing TKA, had only 412 patients, and reported the lowest overall 30-day readmission rate at 2.6 percent. Of the remaining two studies, one only examined pediatric patients while the other examined lumbar spine procedures, both of which produced low 30-day readmission rates at 3.9 and 4.4 percent, respectively. As well, both studies drew from the same clinical registry. Therefore, we do not believe that the source from which the data collected has a significant effect on 30-day readmission rate.

We also found that 30-day readmissions were 55 percent more likely in studies with only Medicare patients as compared to studies reporting unrestricted insurance. Our finding suggests that institutions with a higher percentage of Medicare patients are likely to have higher 30-day readmission rates. This is not surprising because Medicare insurance is associated with increased age and comorbidities, both of which are factors that have been independently associated with higher readmission rates [27,29]. Increased 30-day readmission rates may disincentivize hospitals from accepting Medicare patients to avoid penalties. This could be accounted for statistically by adjusting for risk or weighting Medicare patients differently in readmission calculations. Further studies are required to validate this finding, as this study was unable to control for age and socioeconomic status.

Our results indicate that 30-day readmissions are decreasing over time. Studies that began enrollment before 2009 had significantly higher readmission rates than those that began enrollment after 2009. We believe that this demonstrates an example of the Hawthorne effect, where shining light onto this problem has led to systematic changes to reduce 30-day readmissions. Furthermore, reimbursement changes have incentivized hospitals to begin quality improvement programs. In the future, we hope that specific improvement measures will be developed that can be generalized to all hospitals.

Our study has several limitations. First, many factors that have been shown to affect 30-day readmission rates could not be evaluated including diabetes, body mass index, and corticosteroid use [39]. Second, the definition of “30-day readmission” varied slightly across studies. Some measured 30 days from the date of procedure while others measured from the date of discharge. There are also substantial differences in variable definitions and uses of administrative claims databases and clinical registries, which makes combining them for meta-analysis difficult. Similarly, many causes of readmission had unclear definitions. Some groups therefore had overlap while other causes were excluded. Thus, standard definitions would be useful for future studies. Finally, as demonstrated by the inverse funnel plot, there may be missing studies with fewer patients and higher readmission rates. This is likely because researchers may not publish their institution's high readmission rate.

All publications included in this analysis were retrospective observational studies. While most were prognostic level II studies, which are considered moderate evidence, the conclusions drawn in this analysis are limited by this study type. This highlights a need for randomized controlled trials in orthopedic readmission literature. Over 97 percent of the patients in this meta-analysis were from either spine or arthroplasty studies. This may limit the generalizability of the results to all of orthopedics, however the 30-day readmission rates of those two subspecialties did not significantly differ from the 30-day readmission rate found in studies reporting on all orthopedic subspecialties. One study, Cullen et al., met inclusion criteria, yet the study was completed six years prior to any other study. Sensitivity analysis, however, determined that exclusion of this study would not have significantly changed the results. Finally, few studies have been able to track readmission to outside hospitals and most did not state if they tracked outside hospital admissions. At the time of this submission, 14 of the 20 authors that we contacted regarding unclear inclusion of outside hospitals replied. In total, five of the 24 papers presented in this analysis followed outside hospital readmissions. Even fewer studies (none presented in this analysis) have quantified the frequency of 30-day readmission to the same hospital versus another hospital. This information could be valuable to quality improvement initiatives.

With the data presented in this study, institutions now have a benchmark to which they can compare their 30-day readmission rates. We have also shown however, that there are factors such as subspecialty and patient insurance that are correlated with increased 30-day readmission rates. Thus, these factors should be accounted for when examining 30-day readmission rates. Additionally, the association between patient comorbidities and readmission, which have been demonstrated in other studies and confirmed here, should also be considered.

## Supporting Information

S1 Table Full-text exclusions. Articles reviewed in full that were not included in analysis based on exclusion criteria.
(DOCX)

S2 Table QUIPS analysis. Quality and bias assessment.
(DOCX)

S1 PRISMA Checklist PRISMA Checklist. (DOC)
