# Supplementary material for: Thirty-Day Readmission Rates in Orthopedics: A Systematic Review and Meta-Analysis
Source: PLoS One. 2015 Apr 17;10(4):e0123593. doi: 10.1371/journal.pone.0123593 (PMC4401733; doi:10.1371/journal.pone.0123593)
Supplement: S1 Table — Articles reviewed in full that were not included in analysis based on exclusion criteria. (DOCX) [file pone.0123593.s001.docx]

| S1 Table. Excluded Studies. | |
| --- | --- |
| Article Citation | Reason for Exclusion |
| Miric A, Inacio MC, Kelly MP, Namba RS. Can total knee arthroplasty be safely performed among nonagenarians? An evaluation of morbidity and mortality within a total joint replacement registry. J Arthroplasty. 2014 Aug;29(8):1635-8. | Patients were subgrouped (only included patients over the age of 90) |
| Robbins CE, Casey D, Bono JV, Murphy SB, Talmo CT, et al. A multidisciplinary total hip arthroplasty protocol with accelerated postoperative rehabilitation: does the patient benefit?. Am J Orthop (Belle Mead NJ). 2014 Apr;43(4):178-81. | Tested a specific medical device, surgical technique, or post-operative care protocol |
| Mesko NW, Bachmann KR, Kovacevic D, LoGrasso ME, O'Rourke C, et al. Thirty-day readmission following total hip and knee arthroplasty - a preliminary single institution predictive model. J Arthroplasty. 2014 Aug;29(8):1532-8 | Did not report all-cause 30-day readmission rate |
| Miric A, Inacio MC, Namba RS. The effect of chronic kidney disease on total hip arthroplasty. J Arthroplasty. 2014 Jun;29(6):1225-30. | Patients were subgrouped (only included patients with CKD) |
| Streubel PN, Simone JP, Sperling JW, Cofield R. Thirty and ninety-day reoperation rates after shoulder arthroplasty. J Bone Joint Surg Am. 2014 Feb 5;96(3):e17. | Did not report all-cause 30-day readmission rate |
| Nandyala SV, Marquez-Lara A, Park DK, Hassanzadeh H, Sankaranarayanan S, et al. Incidence, risk factors, and outcomes of postoperative airway management after cervical spine surgery. Spine (Phila Pa 1976). 2014 Apr 20;39(9):E557-63 | Patients were subgrouped (only included patients that required prolonged intubation) |
| Khan SK, Malviya A, Muller SD, Carluke I, Partington PF, et al. Reduced short-term complications and mortality following Enhanced Recovery primary hip and knee arthroplasty: results from 6,000 consecutive procedures. Acta Orthop. 2014 Feb;85(1):26-31. | Tested a specific medical device, surgical technique, or post-operative care protocol |
| Bohensky MA, Ademi Z, deSteiger R, Liew D, Sundararajan V, et al. Quantifying the excess cost and resource utilisation for patients with complications associated with elective knee arthroscopy: a retrospective cohort study. Knee. 2014 Mar;21(2):491-6. | Did not report all-cause 30-day readmission rate |
| Goode AP, Richardson WJ, Schectman RM, Carey TS. Complications, revision fusions, readmissions, and utilization over a 1-year period after bone morphogenetic protein use during primary cervical spine fusions. Spine J. 2014 Sep 1;14(9):2051-9. | Tested a specific medical device, surgical technique, or post-operative care protocol |
| Rappaport DI, Adelizzi-Delany J, Rogers KJ, Jones CE, Petrini ME, et al. Outcomes and costs associated with hospitalist comanagement of medically complex children undergoing spinal fusion surgery. Hosp Pediatr. 2013 Jul;3(3):233-41. | Tested a specific medical device, surgical technique, or post-operative care protocol |
| Bekelis K, Missios S, Kakoulides G, Rahmani R, Simmons N. Selection of patients for ambulatory lumbar discectomy: results from four US states. Spine J. 2014 Sep 1;14(9):1944-50. | Majority were outpatient procedures |
| Veeravagu A, Cole T, Jiang B, Ratliff JK. Revision rates and complication incidence in single- and multilevel anterior cervical discectomy and fusion procedures: an administrative database study. Spine J. 2014 Jul 1;14(7):1125-31. | Did not report all-cause 30-day readmission rate |
| Mioton LM, Buck DW 2nd, Rambachan A, Ver Halen J, Dumanian GA, et al. Predictors of readmission after outpatient plastic surgery. Plast Reconstr Surg. 2014 Jan;133(1):173-80 | Did not report on orthopedic procedures/admissions |
| Ling XW, Howe TS, Koh JS, Wong MK, Ng AC. Preoperative thyroid dysfunction predicts 30-day postoperative complications in elderly patients with hip fracture. Geriatr Orthop Surg Rehabil. 2013 Jun;4(2):43-9. | Patients were subgrouped (elderly patients with thyroid dysfunction) |
| Singh JA, Lu X, Rosenthal GE, Ibrahim S, Cram P. Racial disparities in knee and hip total joint arthroplasty: an 18-year analysis of national Medicare data. Ann Rheum Dis. 2014 Dec;73(12):2107-15. | Started collecting data before year 2000 |
| Wang W, Dudjak LA, Larue EM, Ren D, Scholle C, et al. The influence of goal setting and SmartRoom patient education videos on readmission rate, length of stay, and patient satisfaction in the orthopedic spine population. Comput Inform Nurs. 2013 Sep;31(9):450-6. | Tested a specific medical device, surgical technique, or post-operative care protocol |
| Mehrotra A, Sloss EM, Hussey PS, Adams JL, Lovejoy S, et al. Evaluation of a center of excellence program for spine surgery. Med Care. 2013 Aug;51(8):748-57. | Tested a specific medical device, surgical technique, or post-operative care protocol |
| Schweppe ML, Seyler TM, Plate JF, Swenson RD, Lang JE. Does surgical approach in total hip arthroplasty affect rehabilitation, discharge disposition, and readmission rate?. Surg Technol Int. 2013 Sep;23:219-27. | Tested a specific medical device, surgical technique, or post-operative care protocol |
| Bozic KJ, Ward L, Vail TP, Maze M. Bundled payments in total joint arthroplasty: targeting opportunities for quality improvement and cost reduction. Clin Orthop Relat Res. 2014 Jan;472(1):188-93 | Did not report all-cause 30-day readmission rate |
| Cram P, Ibrahim SA, Lu X, Wolf BR. Impact of alternative coding schemes on incidence rates of key complications after total hip arthroplasty: a risk-adjusted analysis of a national data set. Geriatr Orthop Surg Rehabil. 2012 Mar;3(1):17-26. | Did not report all-cause 30-day readmission rate |
| Bohensky MA, deSteiger R, Kondogiannis C, Sundararajan V, Andrianopoulos N, et al. Adverse outcomes associated with elective knee arthroscopy: a population-based cohort study. Arthroscopy. 2013 Apr;29(4):716-25. | Did not report all-cause 30-day readmission rate |
| Jørgensen CC, Kehlet H. Role of patient characteristics for fast-track hip and knee arthroplasty. Br J Anaesth. 2013 Jun;110(6):972-80. | Tested a specific medical device, surgical technique, or post-operative care protocol |
| Deyo RA, Martin BI, Ching A, Tosteson AN, Jarvik JG, et al. Interspinous spacers compared with decompression or fusion for lumbar stenosis: complications and repeat operations in the Medicare population. Spine (Phila Pa 1976). 2013 May 1;38(10):865-72. | Tested a specific medical device, surgical technique, or post-operative care protocol |
| Cho SK, Yi JS, Park MS, Hu G, Zebala LP, et al. Hemostatic techniques reduce hospital stay following multilevel posterior cervical spine surgery. J Bone Joint Surg Am. 2012 Nov 7;94(21):1952-8. | Tested a specific medical device, surgical technique, or post-operative care protocol |
| Stefan MS, Pekow PS, Nsa W, Priya A, Miller LE, et al. Hospital performance measures and 30-day readmission rates. J Gen Intern Med. 2013 Mar;28(3):377-85 | Did not report on orthopedic procedures/admissions |
| Nacke E, Ramos N, Stein S, Hutzler L, Bosco JA 3rd. When do readmissions for infection occur after spine and total joint procedures?. Clin Orthop Relat Res. 2013 Feb;471(2):569-73. | Did not report all-cause 30-day readmission rate |
| Heidari N, Jehan S, Alazzawi S, Bynoth S, Bottle A, et al. Mortality and morbidity following hip fractures related to hospital thromboprophylaxis policy. Hip Int. 2012 Jan-Feb;22(1):13-21. | Tested a specific medical device, surgical technique, or post-operative care protocol |
| Siracuse JJ, Odell DD, Gondek SP, Odom SR, Kasper EM, et al. Health care and socioeconomic impact of falls in the elderly. Am J Surg. 2012 Mar;203(3):335-8; discussion 338. | Patients were subgrouped (elderly with recent fall) |
| McNair PD, Luft HS. Enhancing Medicare's hospital-acquired conditions policy to encompass readmissions. Medicare Medicaid Res Rev. 2012;2(2) | Did not report all-cause 30-day readmission rate |
| SooHoo NF, Farng E, Krenek L, Zingmond DS. Complication rates following operative treatment of calcaneus fractures. Foot Ankle Surg. 2011 Dec;17(4):233-8. | Did not report all-cause 30-day readmission rate |
| Wilson R, Lewis SA, Dicianno BE. Targeted preventive care may be needed for adults with congenital spine anomalies. PM R. 2011 Aug;3(8):730-8. | Patients were subgrouped (spina bifida) |
| Huddleston JI, Wang Y, Uquillas C, Herndon JH, Maloney WJ. Age and obesity are risk factors for adverse events after total hip arthroplasty. Clin Orthop Relat Res. 2012 Feb;470(2):490-6 | Did not report all-cause 30-day readmission rate |
| Gregersen M, Zintchouk D, Borris LC, Damsgaard EM. A geriatric multidisciplinary and tailor-made hospital-at-home method in nursing home residents with hip fracture. Geriatr Orthop Surg Rehabil. 2011 Jul;2(4):148-54. | Patients were subgrouped (nursing home residents with hip fracture) |
| van Diepen S, Bakal JA, McAlister FA, Ezekowitz JA. Mortality and readmission of patients with heart failure, atrial fibrillation, or coronary artery disease undergoing noncardiac surgery: an analysis of 38 047 patients. Circulation. 2011 Jul 19;124(3):289-96. | Did not report on orthopedic procedures/admissions |
| Cahill KS, Chi JH, Groff MW, McGuire K, Afendulis CC, et al. Outcomes for single-level lumbar fusion: the role of bone morphogenetic protein. Spine (Phila Pa 1976). 2011 Dec 15;36(26):2354-62. | Tested a specific medical device, surgical technique, or post-operative care protocol |
| Jameson SS, Dowen D, James P, Serrano-Pedraza I, Reed MR, et al. Complications following anterior cruciate ligament reconstruction in the English NHS. Knee. 2012 Jan;19(1):14-9. | Did not report all-cause 30-day readmission rate |
| Kates SL, Mendelson DA, Friedman SM. Co-managed care for fragility hip fractures (Rochester model). Osteoporos Int. 2010 Dec;21(Suppl 4):S621-5. | Tested a specific medical device, surgical technique, or post-operative care protocol |
| Bozic KJ, Maselli J, Pekow PS, Lindenauer PK, Vail TP, et al. The influence of procedure volumes and standardization of care on quality and efficiency in total joint replacement surgery. J Bone Joint Surg Am. 2010 Nov 17;92(16):2643-52. | Tested a specific medical device, surgical technique, or post-operative care protocol |
| Press MJ, Silber JH, Rosen AK, Romano PS, Itani KM, et al. The impact of resident duty hour reform on hospital readmission rates among Medicare beneficiaries. J Gen Intern Med. 2011 Apr;26(4):405-11. | Tested a specific medical device, surgical technique, or post-operative care protocol |
| Husted H, Otte KS, Kristensen BB, Ørsnes T, Wong C, et al. Low risk of thromboembolic complications after fast-track hip and knee arthroplasty. Acta Orthop. 2010 Oct;81(5):599-605. | Tested a specific medical device, surgical technique, or post-operative care protocol |
| Gregersen M, Zintchouk D, Hougaard K, Krogshede A, Almasi F, et al. [Interdisciplinary geriatric intervention among nursing home residents with hip fracture reduces mortality]. Ugeskr Laeger. 2010 Jun 21;172(25):1902-7. | Patients were subgrouped (nursing home residents with hip fracture) |
| Friedman SM, Mendelson DA, Bingham KW, Kates SL. Impact of a comanaged Geriatric Fracture Center on short-term hip fracture outcomes. Arch Intern Med. 2009 Oct 12;169(18):1712-7. | Tested a specific medical device, surgical technique, or post-operative care protocol |
| Shi HY, Khan M, Culbertson R, Chang JK, Wang JW, et al. Health-related quality of life after total hip replacement: a Taiwan study. Int Orthop. 2009 Oct;33(5):1217-22. | Started collecting data before year 2000 |
| Batsis JA, Naessens JM, Keegan MT, Huddleston PM, Wagie AE, et al. Resource utilization of total knee arthroplasty patients cared for on specialty orthopedic surgery units. J Hosp Med. 2008 May;3(3):218-27. | Did not report all-cause 30-day readmission rate |
| Seah VW, Singh G, Yang KY, Yeo SJ, Lo NN, et al. Thirty-day mortality and morbidity after total knee arthroplasty. Ann Acad Med Singapore. 2007 Dec;36(12):1010-2. | Did not report all-cause 30-day readmission rate |
| Wilson NC, Stott NS. Paediatric femoral fractures: factors influencing length of stay and readmission rate. Injury. 2007 Aug;38(8):931-6. | Fewer than 100 patients |
| Husted H, Hansen HC, Holm G, Bach-Dal C, Rud K, et al. [Length of stay in total hip and knee arthroplasty in Danmark I: volume, morbidity, mortality and resource utilization A national survey in orthopaedic departments in Denmark]. Ugeskr Laeger. 2006 May 29;168(22):2139-43. | Did not report all-cause 30-day readmission rate |
| Judge A, Chard J, Learmonth I, Dieppe P. The effects of surgical volumes and training centre status on outcomes following total joint replacement: analysis of the Hospital Episode Statistics for England. J Public Health (Oxf). 2006 Jun;28(2):116-24. | Did not report all-cause 30-day readmission rate |
| Teixeira A, Trinquart L, Raphael M, Bastianic T, Chatellier G, et al. Outcomes in older patients after surgical treatment for hip fracture: a new approach to characterise the link between readmissions and the surgical stay. Age Ageing. 2009 Sep;38(5):584-9 | Did not report all-cause 30-day readmission rate |
| Payo J, Perez-Grueso FS, Fernandez-Baillo N, Garcia A. Severe restrictive lung disease and vertebral surgery in a pediatric population. Eur Spine J. 2009 Dec;18(12):1905-10. | Patients were subgrouped (pediatric patients with severe restrictive lung disease) |
| Hahnel J, Burdekin H, Anand S. Re-admissions following hip fracture surgery. Ann R Coll Surg Engl. 2009 Oct;91(7):591-5 | Did not report all-cause 30-day readmission rate |
| Ng HJ, Lee LH. Trends in prevalence of deep venous thrombosis among hospitalised patients in an Asian institution. Thromb Haemost. 2009 Jun;101(6):1095-9. | Did not report all-cause 30-day readmission rate |
| SooHoo NF, Krenek L, Eagan MJ, Gurbani B, Ko CY, et al. Complication rates following open reduction and internal fixation of ankle fractures. J Bone Joint Surg Am. 2009 May;91(5):1042-9. | Did not report all-cause 30-day readmission rate |
| de Lissovoy G, Fraeman K, Hutchins V, Murphy D, Song D, et al. Surgical site infection: incidence and impact on hospital utilization and treatment costs. Am J Infect Control. 2009 Jun;37(5):387-97. | Did not report all-cause 30-day readmission rate |
| Berger RA, Sanders SA, Thill ES, Sporer SM, Della Valle C. Newer anesthesia and rehabilitation protocols enable outpatient hip replacement in selected patients. Clin Orthop Relat Res. 2009 Jun;467(6):1424-30. | Tested a specific medical device, surgical technique, or post-operative care protocol |
| Mattila K, Hynynen M. Day surgery in Finland: a prospective cohort study of 14 day-surgery units. Acta Anaesthesiol Scand. 2009 Apr;53(4):455-63. | Majority were outpatients |
| Berger RA, Kusuma SK, Sanders SA, Thill ES, Sporer SM. The feasibility and perioperative complications of outpatient knee arthroplasty. Clin Orthop Relat Res. 2009 Jun;467(6):1443-9. | Majority were outpatients |
| Tow BP, Chua BS, Fook-Chong S, Howe TS. Concurrent fractures of the hip and wrist: a matched analysis of elderly patients. Injury. 2009 Apr;40(4):385-7. | Fewer than 100 patients |
| Ruiz-Suarez M, Barber FA. Postoperative pain control after shoulder arthroscopy. Orthopedics. 2008 Nov;31(11):1130. | Did not report all-cause 30-day readmission rate |
| Schneider M, Kawahara I, Ballantyne G, McAuley C, Macgregor K, et al. Predictive factors influencing fast track rehabilitation following primary total hip and knee arthroplasty. Arch Orthop Trauma Surg. 2009 Dec;129(12):1585-91. | Fewer than 100 patients |
| Bini SA, Fithian DC, Paxton LW, Khatod MX, Inacio MC, et al. Does discharge disposition after primary total joint arthroplasty affect readmission rates?. J Arthroplasty. 2010 Jan;25(1):114-7. | Did not report all-cause 30-day readmission rate |
| Mericli AF, Moore JH Jr, Copit SE, Fox JW 4th, Tuma GA. Technical changes in paraspinous muscle flap surgery have increased salvage rates of infected spinal wounds. Eplasty. 2008;8:e50. | Did not report all-cause 30-day readmission rate |
| Shi HY, Khan M, Culbertson R, Chang JK, Wang JW, et al. Health-related quality of life after total hip replacement: a Taiwan study. Int Orthop. 2009 Oct;33(5):1217-22. | Did not report all-cause 30-day readmission rate |
