# Supplementary material for: Thirty-Day Readmission Rates in Orthopedics: A Systematic Review and Meta-Analysis
Source: PLoS One. 2015 Apr 17;10(4):e0123593. doi: 10.1371/journal.pone.0123593 (PMC4401733; doi:10.1371/journal.pone.0123593)
Supplement: S2 Table — Quality and bias assessment. (DOCX) [file pone.0123593.s002.docx]

| S2 Table. Quality Assessment Results Based on QUIPS Tool | | | | | | | | | | | | |
| --- | --- | --- | --- | --- | --- | --- | --- | --- | --- | --- | --- | --- |
|  | | | | | | | **QUIPS Six Domains** | | | | | |
| **Study** | **Patients** | **Readmissions (%)** | **JBJS Prognostic Level** | **Causes** | **Univariate** | **Multivariate** | **Study Participation** | **Study Attrition** | **Prognostic Factor Measurement** | **Outcome Measurement** | **Study Confounding** | **Statistical Analysis and Reporting** |
| Cullen, ARCSE 2006^8^ | 769 | 65 (8.5) | III | No | - | - | High | Moderate | NA | Low | NA | Low |
| Vorhies, CORR 2012^9^ | 4057 | 228 (5.7) | III | No | - | - | Moderate | Low | NA | Low | NA | Low |
| Vorhies, JArth 2011^10^ | 1809 | 123 (6.8) | III | No | - | - | Moderate | Low | NA | Low | NA | Low |
| Wang, Spine 2012^11^ | 343068 | 27102 (7.9) | III | Yes | Yes | - | Moderate | Low | Moderate | Low | Moderate | Low |
| Zmistowski, JBJS 2013^12^ | 10633 | 348 (3.3) | III | Yes | - | Yes | High | Unknown | Low | Low | Low | Low |
| Morris, JAMASurg 2014^13^ | 2273 | 175 (7.7) | III | No | - | - | Moderate | Moderate | NA | Low | NA | Low |
| Schairer, CORR (a) 2014^14^ | 1415 | 61 (4.3) | III | Yes | - | Yes | High | Moderate | Moderate | Low | Low | Low |
| Schairer, CORR (b) 2014^15^ | 1408 | 56 (4.0) | III | Yes | - | Yes | High | Moderate | Moderate | Low | Low | Low |
| Cram, MCP 2012^16^ | 64712 | 5320 (8.2) | III | No | - | - | Moderate | Low | NA | Low | NA | Low |
| Schairer, Spine 2013^7^ | 836 | 116 (13.9) | III | Yes | - | Yes | High | Moderate | Low | Low | Low | Low |
| Hoyer, JHM 2014^17^ | 3292 | 273 (8.3) | III | No | - | Yes | Moderate | Moderate | Moderate | Low | Low | Low |
| McCormack, Spine 2012^18^ | 3673 | 156 (4.2) | III | Yes | - | - | High | Moderate | NA | Low | NA | Low |
| Amin, JNSS 2013^19^ | 5780 | 281 (4.9) | III | Yes | - | - | High | Moderate | NA | Low | NA | Low |
| Dailey, JBJSAm 2013^20^ | 3261 | 137 (4.2) | III | Yes | - | Yes | Moderate | Unknown | Low | Low | Low | Low |
| Hageman, JOT 2014^21^ | 3452 | 186 (5.4) | III | Yes | - | Yes | High | Moderate | Moderate | Low | Low | Low |
| Bosco, JArth 2014^22^ | 1077 | 57 (5.3) | III | No | - | - | High | Moderate | NA | Low | NA | Low |
| Bosco, JArth 2014^22^ | 1263 | 55 (4.4) | III | No | - | - | High | Moderate | NA | Low | NA | Low |
| Clement, JArth 2013^23^ | 1583 | 103 (6.5) | III | Yes | Yes | - | High | Low | Moderate | Low | Moderate | Low |
| Mesko, JArth 2014^24^ | 2368 | 159 (6.7) | III | No | Yes | - | Moderate | Moderate | Low | Low | Moderate | Low |
| Mesko, JArth 2014^24^ | 1291 | 46 (3.6) | III | No | Yes | - | High | Moderate | Low | Low | Moderate | Low |
| Kim, JNSS 2014^25^ | 7016 | 314 (4.5) | III | No | - | Yes | Moderate | Low | Low | Low | Low | Low |
| Lovecchio, Spine 2014^26^ | 2320 | 59 (2.6) | III | Yes | - | Yes | Moderate | Low | Low | Low | Low | Low |
| Basques, Spine 2014^27^ | 2339 | 87 (3.7) | III | Yes | - | Yes | Moderate | Moderate | Low | Low | Low | Low |
| Pugely, Spine 2014^28^(a) | 2005 | 79 (3.9) | III | No | - | Yes | Moderate | Low | Moderate | Low | Low | Low |
| Pugely, Spine 2014^29^(b) | 15668 | 695 (4.4) | III | Yes | - | Yes | Moderate | Low | Moderate | Low | Low | Low |
| Issa, JKS 2014^6^ | 412 | 8 (2.0) | III | No | - | - | Moderate | Unknown | NA | Low | NA | Low |
